# Supplementary material for: Rheological and Self-Healing Behavior of Hydrogels Synthesized from l-Lysine-Functionalized Alginate Dialdehyde
Source: Polymers (Basel). 2023 Feb 17;15(4):1010. doi: 10.3390/polym15041010 (PMC9959054; doi:10.3390/polym15041010)
Supplement: Supplementary file 1 [file polymers-15-01010-s001.zip › polymers-2138414-supplementary.pdf]

Supplementary materials

Rheological and Self-Healing Behavior of Hydrogels Synthesized from L-Lysine-Functionalized Alginate Dialdehyde

Arlina Prima Putri [1,2], Ranjita K. Bose [1], Mochamad Chalid [2] and Francesco Picchioni [1,*]

[1] Department of Chemical Engineering—Product Technology, University of Groningen, Nijenborgh 4, 9747 AG Groningen, The Netherlands
[2] Metallurgical and Material Engineering Department, Universitas Indonesia, Depok 16424, Indonesia
* Correspondence: f.picchioni@rug.nl

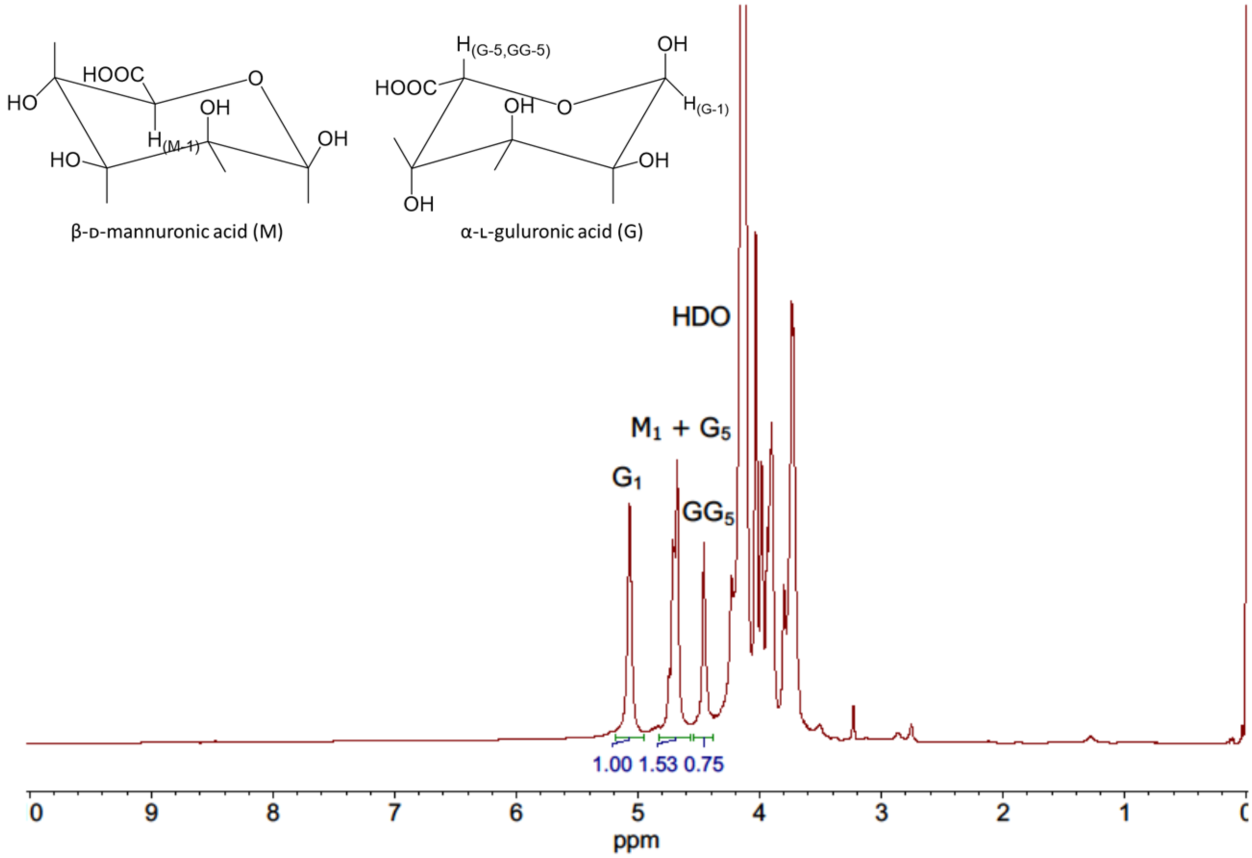

Figure S1. ^{1}H NMR spectra of sodium alginate in D_2O with M/G ratio 1.29, recorded at 90°C.

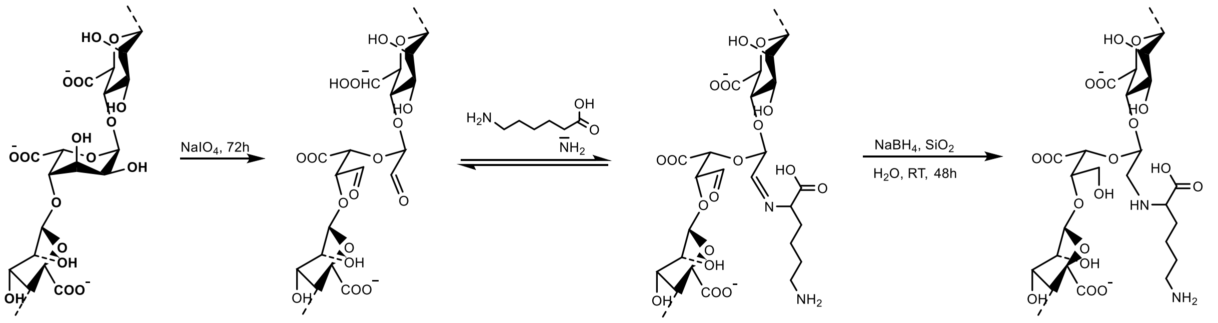

Figure S2. Periodate oxidation of alginate (represented by MGM fragment) followed by reductive amination ($NaBH_4$, SiO_2) to couple L-lysine.

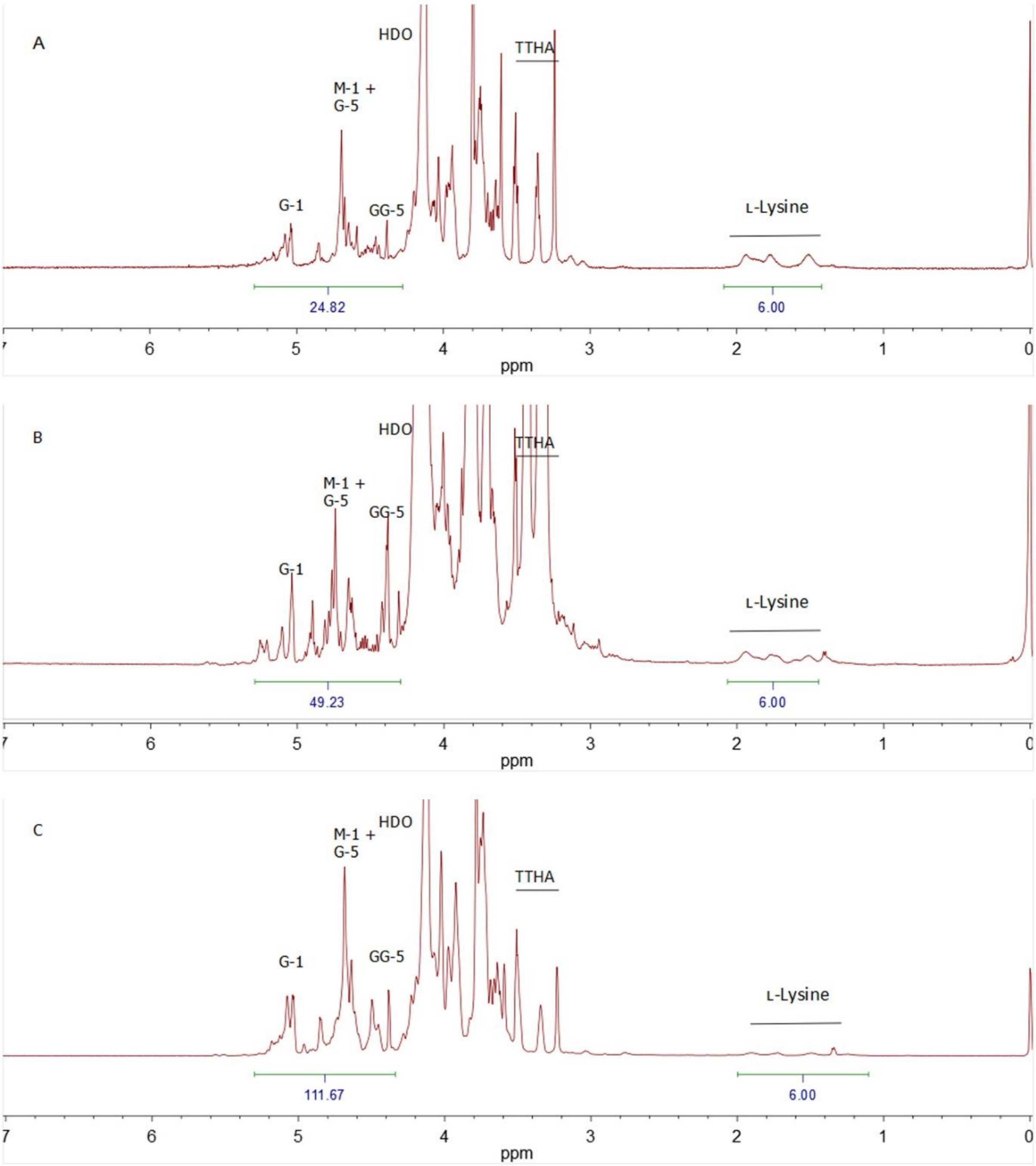

Figure S3. ^{1}H NMR spectra of L-lysine functionalized ADA with a different molar ratio of the substrates (ADA: L-lysine) (A) 2:1 (B) 1:1 and (C) 1:2, recorded at 90°C.

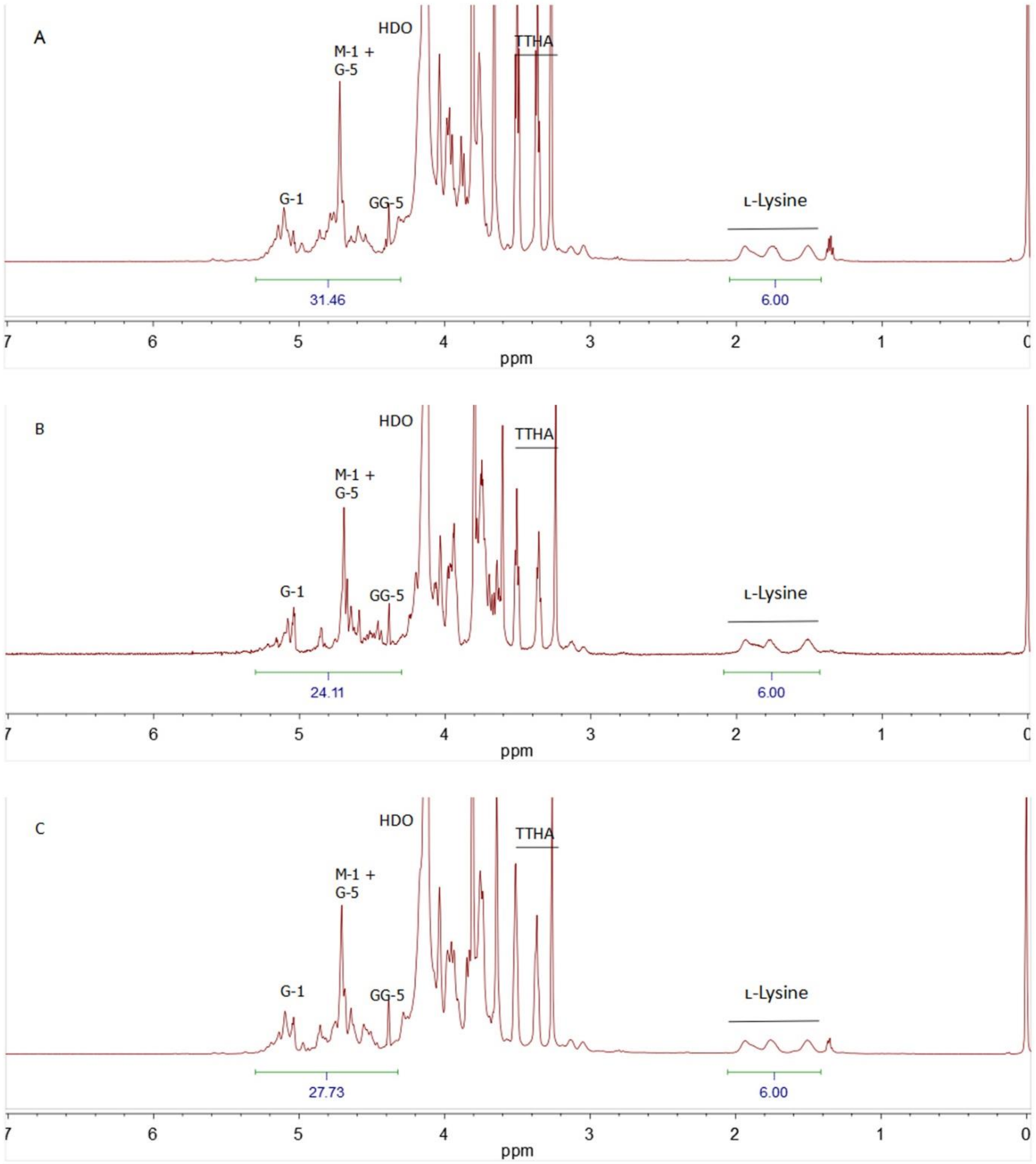

Figure S4. ^{1}H NMR spectra of L-lysine functionalized ADA with a different amount of SiO_2 (A) 2 % (B) 6 % and (C) 10% (w/v), recorded at 90°C.

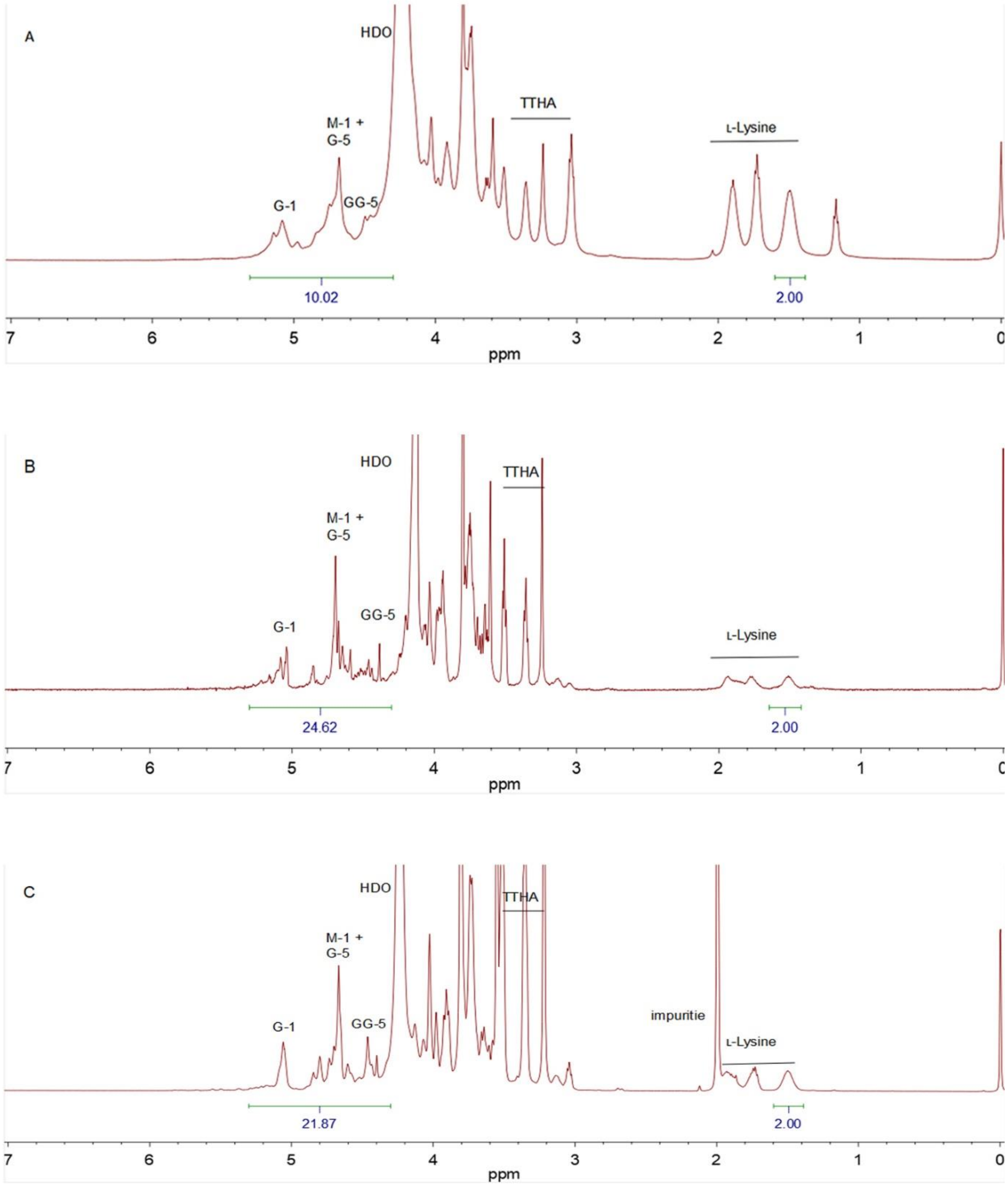

Figure S5. ^{1}H NMR spectra of L-lysine functionalized ADA with a different molar ratio of the substrates to reduction agent $NaBH_4$ (A) 1:1 (B) 1:3 and (C) 1:5, recorded at 90°C.

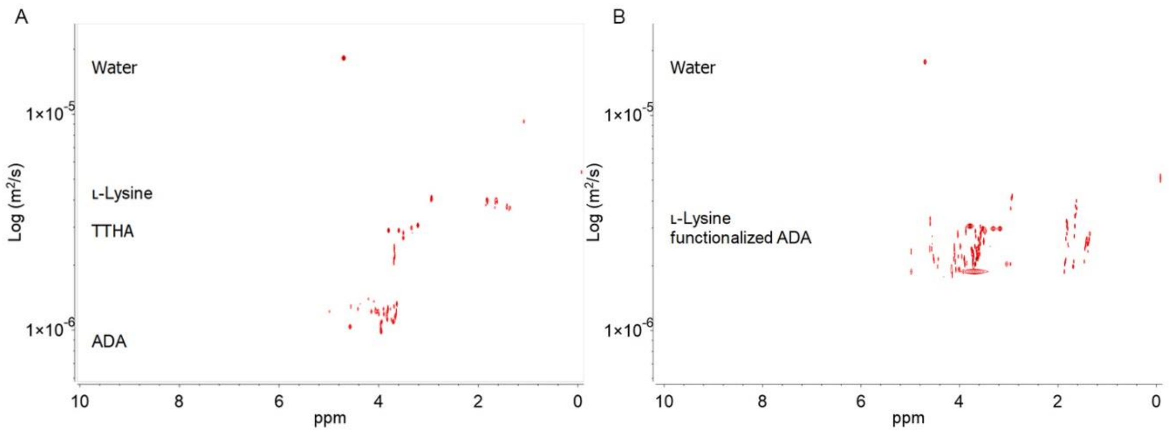

Figure S6. DOSY-spectra of a mixture of L-lysine functionalized ADA with a different molar ratio of the substrates to reduction agent $NaBH_4$ (A) 1:1 (B) 1:5 in D_2O recorded at 25 °C. .

Document: 2021-01-14 (varioMICRO) from: –.–.– (modified)

Elemental Analysis
varioMICRO CHNS

Text report

No.	Name	N [%]
28	RB_Lys_090920	2.47
29	RB_Lys_090920	2.47

Name: eassuperuser, Access: VarioMICRO superuser ma jan 18 12:03:46 2021

varioMICRO V4.0.15 (e2f937f17)2018-08-10, CHNS Mode, Ser. No.: 15171045
Elementar Analysensysteme GmbH

Page 1 (of 1)

Figure S7. Elemental analysis result.

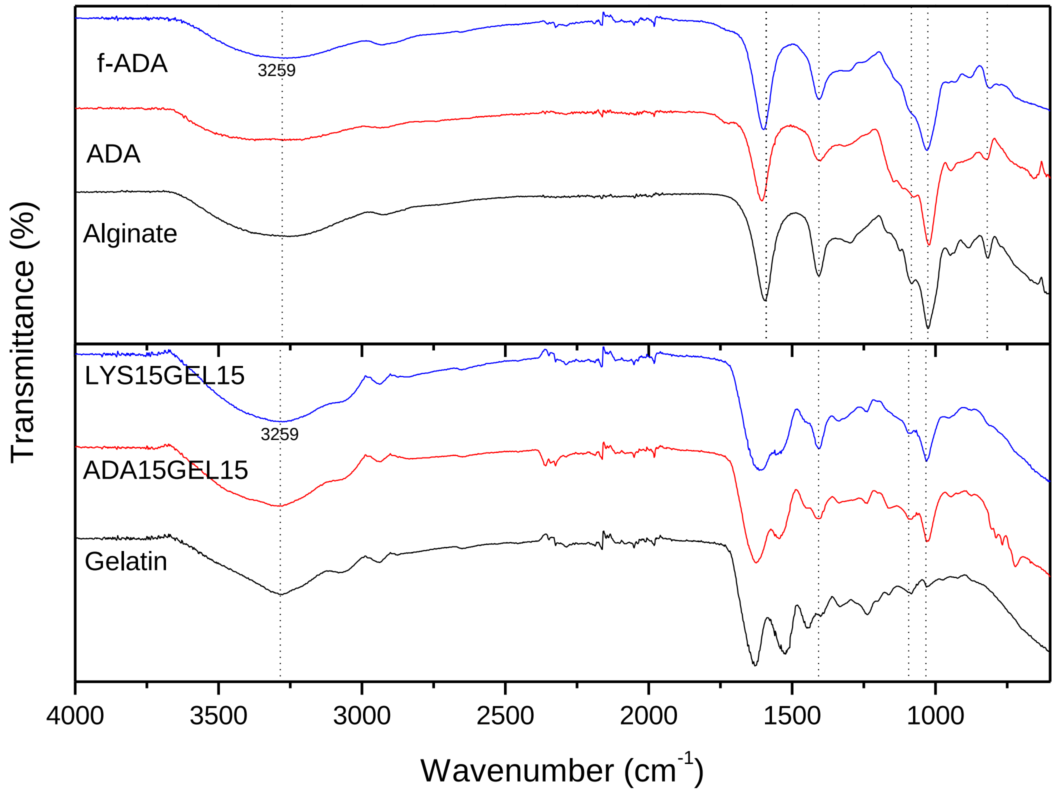

Figure S8. ATR-FTIR spectra of sodium alginate, ADA, and L-lysine functionalized ADA (Top) and ADA15GEL15, LYS15GEL15, and gelatin (Below). The dash represents the alginate characteristic absorption peak.

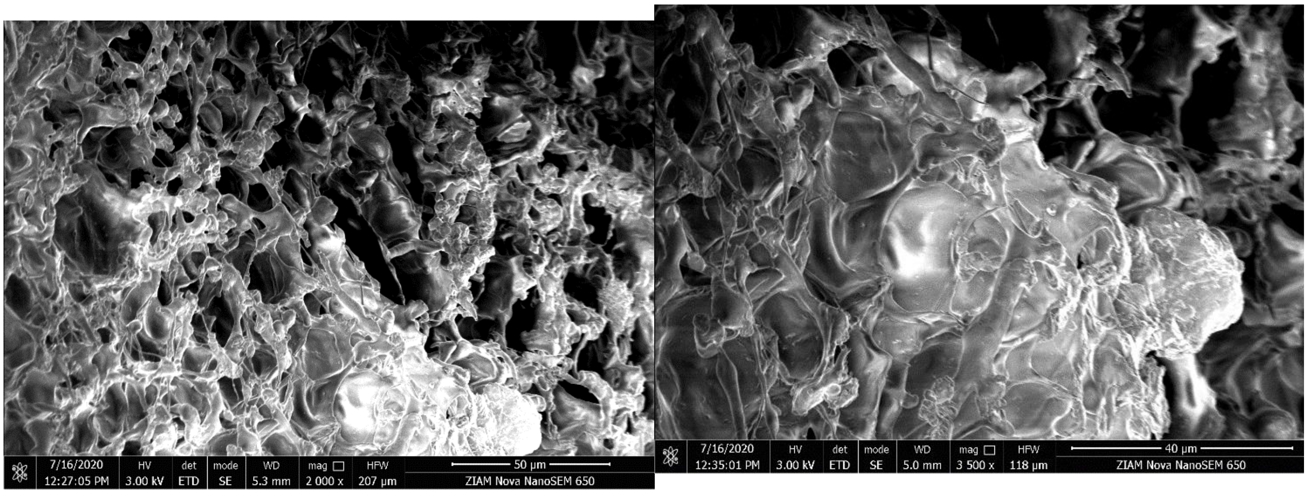

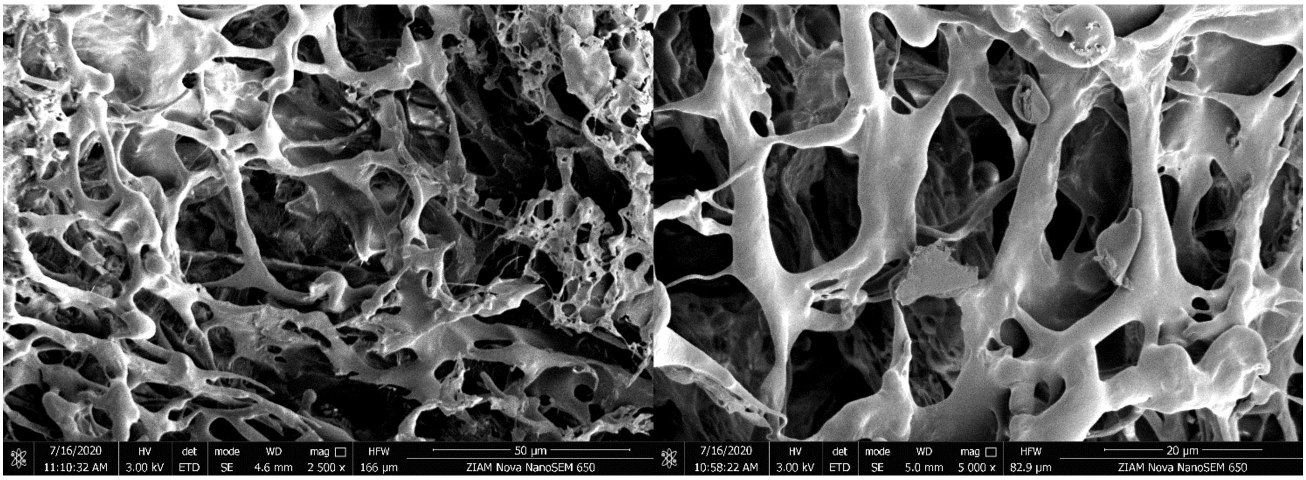

Figure S9. Scanning electron microscopy (SEM) image of ADA (top) and f-ADA (bottom) hydrogels.

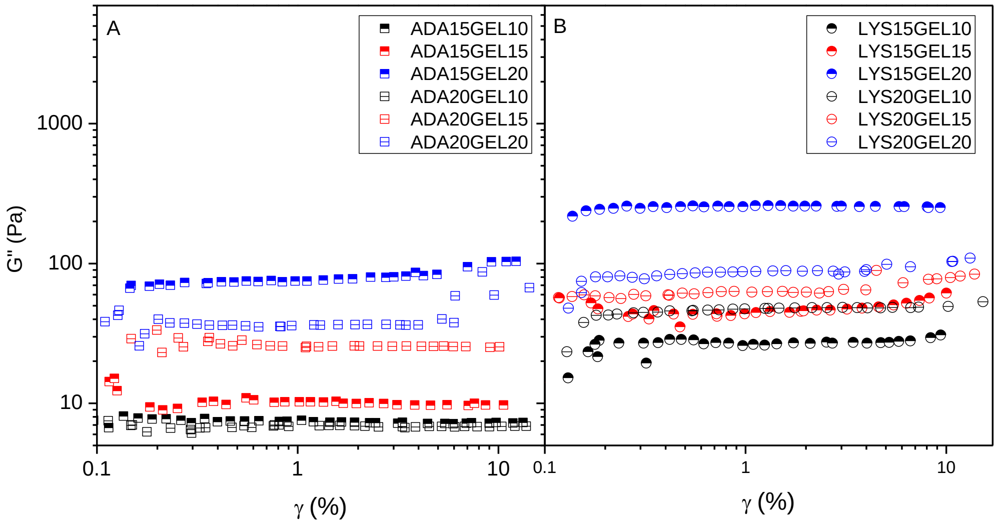

Figure S10. Viscous modulus (G'') of alginate derivate hydrogels samples crosslinked with 10 (black), 15 (red) and 20 (blue) (% w/v) of gelatin. 15 % (w/v) of ADA and 20 % (w/v) of ADA (A). 15 % (w/v) of L-lysine functionalized ADA and 20 % (w/v) of L-lysine functionalized ADA (B).

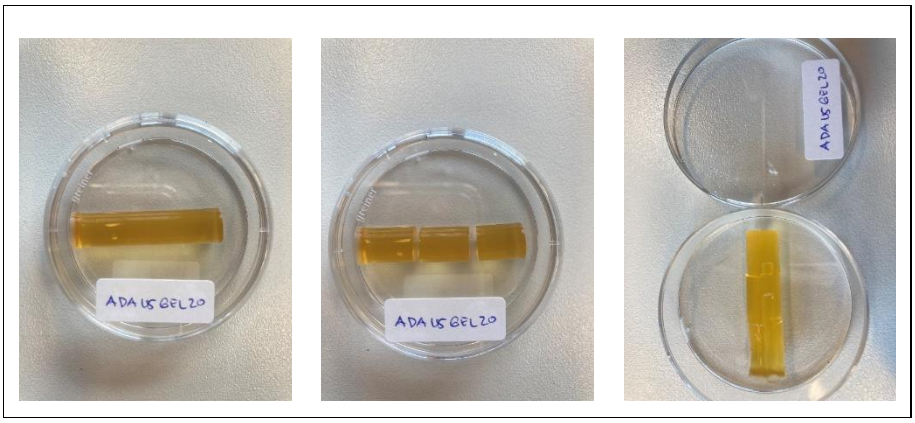

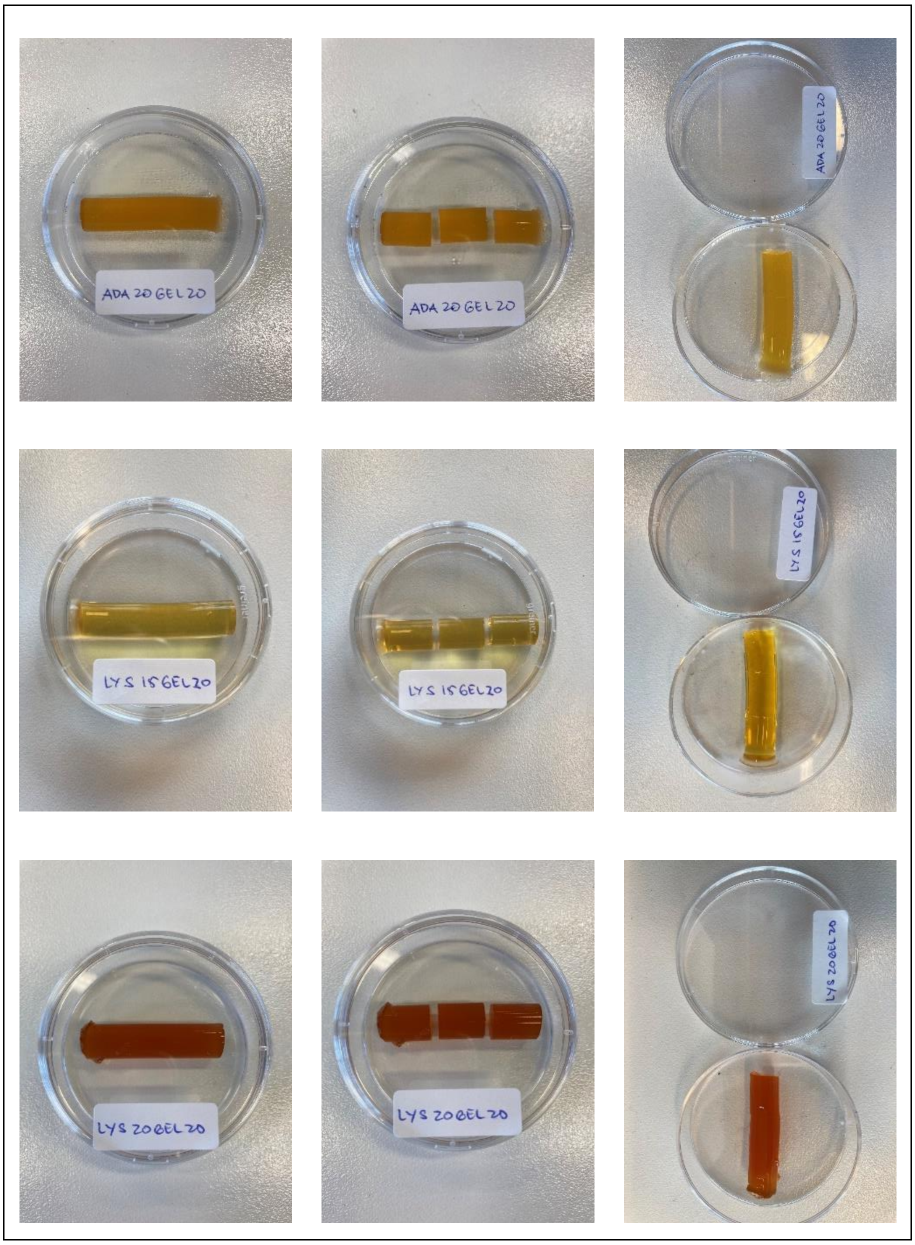

Figure S11. Visualization of the self-healing of monolithic hydrogels. Fresh made monolith before and after cut into three pieces (left and middle). Self-healed hydrogels after seven days resting (right).

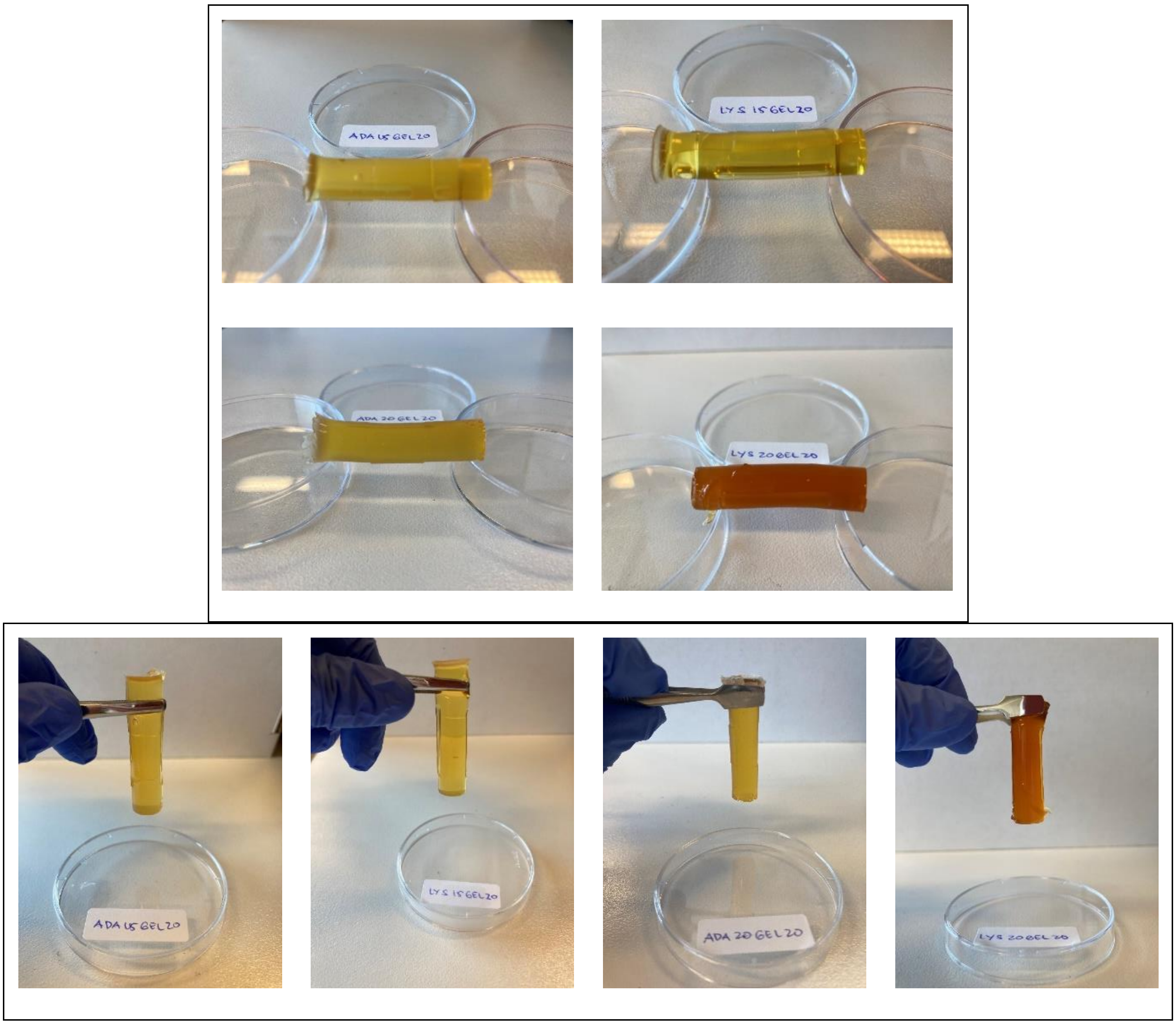

Figure S12. Photographs showing resistance of healed hydrogels to mechanical stresses: bridge formation (left) and hanging (right).
